# Seasonal Succession of Free-Living Bacterial Communities in Coastal Waters of the Western Antarctic Peninsula

**DOI:** 10.3389/fmicb.2016.01731

**Published:** 2016-11-03

**Authors:** Catherine M. Luria, Linda A. Amaral-Zettler, Hugh W. Ducklow, Jeremy J. Rich

**Affiliations:** ^1^Department of Ecology and Evolutionary Biology, Brown University, ProvidenceRI, USA; ^2^The Josephine Bay Paul Center for Comparative Molecular Biology and Evolution, Marine Biological Laboratory, Woods HoleMA, USA; ^3^Department of Earth, Environmental and Planetary Sciences, Brown University, ProvidenceRI, USA; ^4^Department of Earth and Environmental Sciences, Lamont-Doherty Earth Observatory of Columbia University, PalisadesNY, USA; ^5^School of Marine Sciences and Darling Marine Center, University of Maine, WalpoleME, USA

**Keywords:** 16S rRNA gene, ecological succession, Antarctica, bacterial production, bacterial community composition, *Polaribacter*, pelagibacter ubique (SAR11), Rhodobacteraceae

## Abstract

The marine ecosystem along the Western Antarctic Peninsula undergoes a dramatic seasonal transition every spring, from almost total darkness to almost continuous sunlight, resulting in a cascade of environmental changes, including phytoplankton blooms that support a highly productive food web. Despite having important implications for the movement of energy and materials through this ecosystem, little is known about how these changes impact bacterial succession in this region. Using 16S rRNA gene amplicon sequencing, we measured changes in free-living bacterial community composition and richness during a 9-month period that spanned winter to the end of summer. Chlorophyll *a* concentrations were relatively low until summer when a major phytoplankton bloom occurred, followed 3 weeks later by a high peak in bacterial production. Richness in bacterial communities varied between ~1,200 and 1,800 observed operational taxonomic units (OTUs) before the major phytoplankton bloom (out of ~43,000 sequences per sample). During peak bacterial production, OTU richness decreased to ~700 OTUs. The significant decrease in OTU richness only lasted a few weeks, after which time OTU richness increased again as bacterial production declined toward pre-bloom levels. OTU richness was negatively correlated with bacterial production and chlorophyll *a* concentrations. Unlike the temporal pattern in OTU richness, community composition changed from winter to spring, prior to onset of the summer phytoplankton bloom. Community composition continued to change during the phytoplankton bloom, with increased relative abundance of several taxa associated with phytoplankton blooms, particularly *Polaribacter*. Bacterial community composition began to revert toward pre-bloom conditions as bacterial production declined. Overall, our findings clearly demonstrate the temporal relationship between phytoplankton blooms and seasonal succession in bacterial growth and community composition. Our study highlights the importance of high-resolution time series sampling, especially during the relatively under-sampled Antarctic winter and spring, which enabled us to discover seasonal changes in bacterial community composition that preceded the summertime phytoplankton bloom.

## Introduction

Strong seasonal patterns in the marine ecosystem west of the Antarctic Peninsula (WAP) provide a natural experiment to assess how bacteria respond over time to changes in both biotic and abiotic factors. The Antarctic spring brings about a cascade of environmental changes, including light-driven modification of dissolved organic matter (DOM), sea ice melting and retreat, warmer water temperatures and stratification of the water column. These physical changes trigger phytoplankton blooms that support large stocks of upper level consumers (Smetacek and Nicol, 2005) and represent a potentially important sink for atmospheric CO_2_ (Arrigo et al., 2008).

In the water column, some bacterial taxa are adapted to colonize and attach to particles through surface adhesion and gliding motility, while other taxa are more adapted toward life as free-living cells (Dang and Lovell, 2016). This is reflected in differences in community composition between the particle attached and free-living communities (DeLong et al., 1993; Ortega-Retuerta et al., 2013; Rieck et al., 2015). Particle attached bacteria play an important role in the initial degradation of particulate matter, hydrolyzing polymers and releasing smaller molecules that can diffuse away from particles and be utilized by free-living bacteria (Stocker, 2012; Dang and Lovell, 2016). While particle attached bacteria may have higher specific activity, free-living cells generally contribute more to overall bacterial activity in the water column due to greater overall cell abundance, but there are exceptions (Iriberri et al., 1987; Turley and Mackie, 1994; Rieck et al., 2015). Therefore, studies that focus on free-living cells describe an important part of the bacterial community but not the entire community (Teeling et al., 2012; Williams et al., 2013; Sunagawa et al., 2015).

Bacterial interactions with phytoplankton contribute to ecosystem function in multiple ways in both the WAP and the global ocean (Cole, 1982; Croft et al., 2005; Sher et al., 2011). Sustained primary production is partly dependent upon the microbial loop, in which particle attached and free-living heterotrophic bacteria degrade DOM and are consumed by bacterivores, thereby recycling nutrients (Azam et al., 1983). Studies in marine ecosystems indicate that bacterial growth is frequently dependent on phytoplankton-derived DOM (Church et al., 2000; Morán et al., 2001, 2002; Piquet et al., 2011; Ducklow et al., 2012; Kim et al., 2014). Phytoplankton derived carbon likely influences the succession of bacterial communities as various bacterial taxa differ in their ability to degrade phytoplankton derived DOM and particulate detritus (Kerkhof et al., 1999; Pinhassi et al., 2004; Teeling et al., 2012). Lower bacterial diversity, in terms of both richness and evenness, accompanies seasonal changes, reflecting an increase in abundance of relatively few bacterial taxa (Gilbert et al., 2012; Ladau et al., 2013).

Certain groups of bacteria (e.g., Flavobacteria and Rhodobacteraceae) increase in abundance during phytoplankton blooms, while other groups such as *Pelagibacter* are better adapted to the free-living state and non-bloom conditions (Williams et al., 2013; Buchan et al., 2014; Voget et al., 2015). Flavobacteria, described as ‘first responders’ to phytoplankton blooms, break down complex organic matter by direct attachment and exoenzymatic attack of phytoplankton cells and phytoplankton-derived detrital particles (Williams et al., 2013). An abundant genus of Flavobacteria in the bacterioplankton is *Polaribacter*, which possess traits consistent with a life-strategy of particle attachment and polymer degradation (Fernández-Gómez et al., 2013). However, *Polaribacter* is metabolically flexible and also abundant in the free-living community, indicating that its ecological niche extends beyond particle attachment (Smith et al., 2013; Williams et al., 2013). Flammeovirgaceae, a family within Bacteroidetes, has been associated with the degradation of algal-derived polysaccharides (Chan et al., 2015; Liu et al., 2015). Members of Rhodobacteraceae are often found in close association with phytoplankton blooms in either the particle attached or free-living part of the community. They generally use small molecular weight substrates, including the degradation products produced by Flavobacteria (Pinhassi et al., 2004; West et al., 2008; Wemheuer et al., 2015).

Studies of bacterial seasonal succession have emphasized the role of bacteria as degraders of labile organic matter (Teeling et al., 2012; Moran, 2015; Needham and Fuhrman, 2016). However, there is increasing evidence that bacterial interactions with phytoplankton may influence the development of phytoplankton blooms themselves through bacterial production of key vitamins, chelating agents, or hormones that stimulate or impede phytoplankton growth (Amin et al., 2012, 2015; Prieto et al., 2015; Wang et al., 2016). Bacterial succession prior to the onset of phytoplankton blooms has been arguably under-studied (Moran, 2015; Needham and Fuhrman, 2016).

Previous analyses in the WAP, based either on community fingerprinting techniques (i.e., denaturing gradient gel electrophoresis) over one or more seasons, or on high-throughput DNA sequencing from only few mid-winter and mid-summer sampling dates, hint at a relationship between bacterial community succession and phytoplankton blooms similar to that observed in more temperate regions (Murray et al., 1998; Murray and Grzymski, 2007; Grzymski et al., 2012; Luria et al., 2014). However, the intervening time period between winter and summer is severely under-sampled in the WAP, as it is throughout the Southern Ocean, making comparisons to other systems difficult. Our objective was to obtain a new high-resolution seasonal time-series of bacterial properties to determine how free-living bacterial succession proceeds during the dynamic Antarctic winter to summer transition. We hypothesized that a phytoplankton bloom would trigger bacterial succession as has been demonstrated previously in temperate regions (e.g., Teeling et al., 2012). Our findings on bacterial community succession provide new evidence of coupling between phytoplankton and bacterial blooms.

## Materials and Methods

### Field Sampling and Contextual Data

Seawater samples were collected from coastal surface waters at Palmer Station, on the west coast of the Antarctic Peninsula at one to 2 week intervals beginning in the austral mid-winter (July 2013) and ending in late summer (March 2014). Samples were drawn directly from a seawater intake located at a depth of 6 m, 16 m from the station. Triplicate 20-L samples were collected in acid-washed Nalgene carboys and immediately transferred to the laboratory for processing. All processing took place in a 0°C cold room to maintain initial water temperature.

Each 20-L carboy was sub-sampled for dissolved nutrients (phosphate, silicate, and nitrate), particulate organic carbon and nitrogen (POC and PON), chlorophyll *a* (chl *a*), and bacterial abundance and bacterial production measurements. Samples were collected and processed according to Palmer LTER standard protocols^1^. Briefly, nutrient samples were filtered through combusted 0.7-μm glass fiber filters (Whatman, GE Healthcare Life Sciences, Piscataway, NJ, USA) and frozen at -80°C until analysis on a SEAL AutoAnalyzer 3 (data available at doi:10.6073/pasta/e893d71c5586769731875d49fde21b1d; Ducklow, 2016). POC and PON samples were collected on combusted 0.7-μm glass fiber filters from 1 to 3 L of seawater and were frozen at -80°C until analysis via combustion using a Perkin Elmer 2400 Series II CHNS/O Analyzer. Chl *a* samples, as with POC and PON, were collected on 0.7-μm glass fiber filters from 1 to 3 L of seawater and were assayed fluorometrically using acetone extracts (data available at doi: 10.6073/pasta/012de0cf7d1f00951b7289037a3a4c19; Schofield and Vernet, 2016). Bacterial abundance samples were analyzed by flow cytometry following the protocol of Gasol and Del Giorgio (2000), with SYBR^®^ Green I nucleic acid staining (Invitrogen, Carlsbad, CA, USA) on an Accuri C6 flow cytometer (BD Biosciences, San Jose, CA) (data available at doi:10.6073/pasta/012de0cf7d1f00951b7289037a3a4c19; Ducklow et al., 2016). Bacterial production rates were derived from rates of ^3^H-leucine incorporation (Ducklow et al., 2012).

Samples for bacterial community composition were collected via gentle vacuum filtration (-0.5 bar vacuum) of approximately 2–6 L of seawater through successive 3.0-μm polycarbonate (EMD Millipore, Billerica, MA, USA) and 0.22-μm polyethersulfone (EMD Millipore, Billerica, MA, USA) filters. Filters were flash-frozen with liquid N_2_ and stored at -80°C until further processing.

### 16S rRNA Gene Library Generation

Bacteria that passed through 3.0 μm pore-size filters and were retained on 0.22 μm pore-size filters were analyzed, and therefore our focus is on the free-living bacterial community composition and not the particle attached component. DNA was extracted from cells captured on the 0.22-μm pore size filters using a DNeasy Plant Mini Kit (Qiagen, Valencia, CA, USA) with an additional bead-beating step. Initially, each filter was cut into small pieces and placed into a tube along with 0.1-mm diameter silica beads. After incubation with AP1 and RNase as in the manufacturer’s protocol, samples were vigorously vortexed for 1 min to lyse cells. DNA extraction was then completed according to the manufacturer’s protocol.

For each DNA sample, the V6 hypervariable region of the bacterial 16S rRNA gene was amplified in two stages following the protocol described previously in Eren et al. (2013). In the first stage, ∼100 bp of the V6 region was amplified in triplicate 33-μl PCR reactions containing 1.0 unit of Platinum Taq Hi-Fidelity Polymerase (Life Technologies, Carlsbad, CA, USA), 1X Hi-Fidelity buffer, 200 μM dNTP PurePeak DNA polymerase mix (Pierce Nucleic Acid Technologies, Milwaukee, WI, USA), 2.0 mM MgCl_2_, 0.06% BSA, 0.2 μM forward and reverse non-fusion primers (Eren et al., 2013), and approximately 10 ng template DNA. The PCR reaction consisted of a 3-min initial denaturation step at 94°C, 25 cycles of 94°C for 30 s, 60°C for 45 s, and 72°C for 60 s, and a final 2-min extension at 72°C. After amplification, triplicate samples were pooled and purified using a MinElute Reaction Cleanup Kit (Qiagen, Valencia, CA, USA) with DNA finally eluted in 10 μl of Qiagen EB buffer.

After quality checks using a Fragment Analyzer (Advanced Analytics, Ames IA), a second fusion PCR reaction was conducted with a set of custom fusion primers consisting of Illumina adaptors, 12 different inline barcodes (forward primers), 8 dedicated indices (reverse primers), and the same V6 primer sequences used in the first round of PCR. The PCR reaction conditions were similar to those described above except that only a single reaction was run for each sample with approximately 2–3 ng purified PCR product as template and 10 reaction cycles. After the final concentration of PCR products was determined using a Qubit 3.0 fluorometer with PicoGreen (LifeTechnologies, Carlsbad, CA, USA), equimolar amounts of each sample were pooled. The 200–240 bp fraction of the sample pool was selected on 1% agarose using a Pippin Prep (SageScience, Beverly, MA, USA) and the final DNA concentration was measured using qPCR (Kapa Biosystems, Woburn, MA, USA) prior to sequencing on one lane of an Illumina HiSeq 1000 cycle paired-end run.

### Data Analysis

Low-quality sequences were filtered from the resulting data by discarding reads without 100% consensus between forward and reverse paired-end sequencing reads (Eren et al., 2013). Observed taxonomic units (OTUs) were clustered using Qiime’s (v 1.9.1) open reference OTU picking with the default UCLUST method, a minimum cluster size of 2, and a 97% similarity threshold and were assigned Greengenes taxonomy (version 13_8) (Caporaso et al., 2010; Edgar, 2010; McDonald et al., 2012). After removing OTUs classified as chloroplasts, rarefied libraries were produced by randomly down-sampling to the smallest library size, 43,308 sequences. Subsequent analyses were based on rarefied libraries unless otherwise indicated.

Alpha diversity metrics included the number of OTUs observed in each library, Shannon’s diversity, and Pielou’s evenness, as well as non-parametric Chao–Jost richness estimates based on un-rarefied libraries as implemented in the R package iNEXT (Chao and Jost, 2012; Chao et al., 2014). The beta diversity between samples was visualized with non-metric multidimensional scaling (NMDS) based on Bray-Curtis similarity using the metaMDS function in the vegan R package (Oksanen et al., 2016). NMDS was also used to assess several normalization methods for un-rarefied libraries: converting read numbers to relative abundances, as well as Relative Log Expression (RLE) and Trimmed Mean of *M*-Values (TMM) normalizations as implemented in edgeR (McMurdie and Holmes, 2013).

To identify OTUs with non-random temporal patterns, we first selected OTUs with a relative abundance ≥0.01 in at least three samples. We assessed the seasonality of this subset through local polynomial regression (LOESS) with serial day as the independent variable and relative abundance as the dependent variable, using *r*^2^ > 0.8 as a threshold to identify OTUs with non-random temporal patterns. We used these OTUs to generate a co-occurrence network based on Pearson’s correlation (*r* > 0.5) using the CoNet plugin in Cytoscape (Shannon et al., 2003; Faust et al., 2012). We extracted non-overlapping clusters from this network through k-means clustering using the Cytoscape clusterMaker2 plugin after first selecting a reasonable value for k through the evaluation of a scree plot of within-clusters sum of squares (Supplementary Figure S1; Morris et al., 2011). The resulting network clusters were visualized in Cytoscape. This helped us to identify groups of OTUs with similar temporal trends.

In order to model the relationship between environmental drivers and potentially delayed bacterial responses, we used linear interpolation as implemented in the R package zoo to predict values between sampling dates and create regular time series (Zeileis and Grothendieck, 2005). We then performed stepwise linear regression with forward-backward variable selection as implemented in the R packages dynlm and MASS, minimizing Akaike Information Criterion (Venables and Ripley, 2002; Zeileis, 2016). Regression models were tested for multicollinearity among predictor variables and models with variance inflation factors (VIF) greater than 5 discarded. The variables tested included temperature, inorganic nutrient levels, POC, and PON. To account for delayed bacterial responses, we built distributed lag models, testing 0-, 10-, and 20-day time lags (Jorgenson, 1966). Additional details are given in Supplementary Text S1 and Supplementary Table S1.

The R code used for all data analysis and figure production can be accessed at: https://github.com/cmluria/Palmer-Station-Bacterial-Succession. All of our sequence data are MIMARKS-compliant (Yilmaz et al., 2011) and have been deposited in the NCBI SRA under the accession number SRP091049. Associated MIMARKS-compliant metadata appear in Supplementary Table S2.

## Results

**FIGURE 1:**
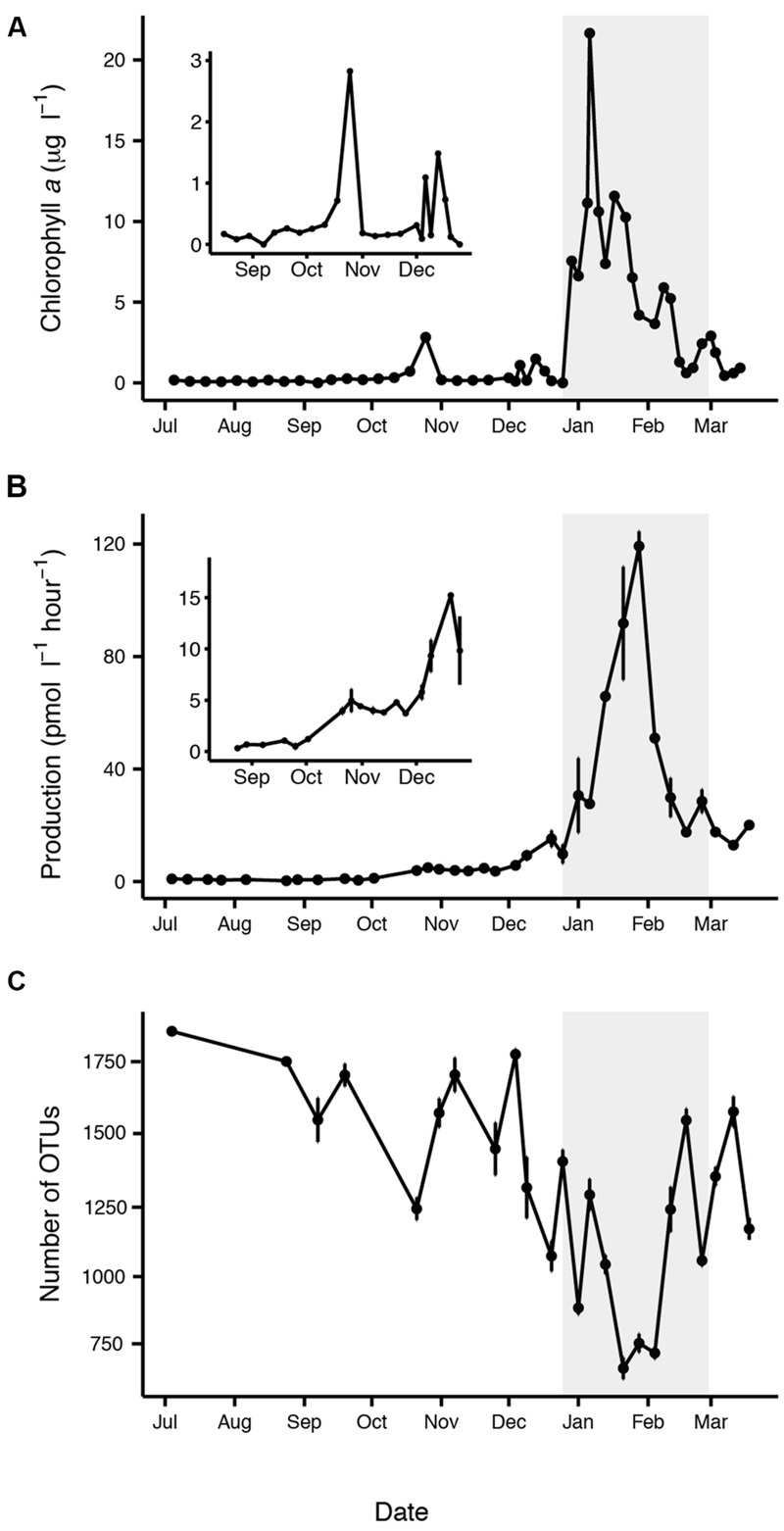
**Characteristics of the summer phytoplankton bloom period (highlighted in gray), based on (A)** increased chl *a*, **(B)** increased bacterial production, and **(C)** decreased free-living bacterial OTU richness. Insets are shown corresponding to the time period before the summer phytoplankton bloom **(A,B)**. Error bars represent standard error for each date.

Our sampling period encompassed changes in day length from ∼4 h of sunlight when we began sampling in July to ∼22 h of sunlight in December. Water temperatures reached a minimum of -1.1°C in August and a maximum of 1.6°C in January (Supplementary Figure S2). Sea ice cover in the Palmer region is variable from year to year; 2013 was a heavy sea ice year with dense sea ice cover during the winter that persisted at significant levels until December (Stammerjohn et al., 2008; Massom et al., 2014). Chl *a*, a proxy for phytoplankton biomass was low (<0.6 μg l^-1^) until October when a brief increase in chl *a* occurred. The primary phytoplankton bloom peaked in summer (early January), based on chl *a*, and then declined towards pre-bloom levels in March (**Figure 1A**). Dissolved inorganic nutrients (phosphate, silicate, and nitrate) were drawn down during the summer bloom, while particulate carbon and nitrogen increased, reflecting phytoplankton production (Supplementary Figure S2). Silicate drawdown was less than nitrate during the summer bloom, suggesting that other phytoplankton groups in addition to diatoms contributed to the bloom. Bacterial production was very low but still detectable from July to October (**Figure 1B**). By January bacterial production increased substantially, peaking about 3 weeks after the peak in chl *a* (**Figures 1A,B**). Our bacterial production rates measured from the seawater intake were similar to those measured at a near shore station (Supplementary Figure S3; data available at doi: 10.6073/pasta/814628d18d4e23753d1164f0bd81095e). Stepwise linear regression revealed that chl *a* with time lags of 0, 10, and 20 days was a good predictor of both bacterial production (*r^2^* = 0.93) and abundance (*r^2^* = 0.62; Supplementary Figure S4; Supplementary Table S1). Several other individual factors (phosphate, silicate, nitrate, POC, and PON) were also significantly correlated with bacterial production (Supplementary Table S1).

In total, 68 samples, spread across 24 sampling dates, were sequenced, yielding 15 million short-read V6 16S rRNA gene sequences (∼43,000–550,000 per library) corresponding to the free-living bacterial community, assigned to 28,857 OTUs. Given recent discussion regarding the statistical validity of library resampling (McMurdie and Holmes, 2014), we assessed different methods to characterize bacterial richness and community composition using un-rarefied sequence libraries. Although the method described by Chao et al. (2014) is intended to estimate true richness based on coverage regardless of sequencing depth, both observed (*p* < 0.001, *r*^2^ = 0.4) and estimated richness (*p* < 0.001, *r*^2^ = 0.35) significantly correlated with library size (Supplementary Figure S5). This may indicate that the species abundance distributions vary widely among our datasets (Gwinn et al., 2016). Likewise, NMDS based on un-rarefied sequence libraries showed the influence of initial library size even after relative abundance, RLE, and TMM normalizations (Supplementary Figure S6). For these reasons, all subsequent analyses relied on libraries rarefied to the smallest library size of 43,308 sequences.

Observed richness of the free-living bacterial community varied significantly between midwinter and midsummer (*p* < 0.0001), ranging from a maximum of approximately 1800 OTUs in July to a minimum of approximately 700 OTUs in January (**Figure 1**). On average, Chao–Jost estimated richness was four times greater than observed richness. Richness was negatively correlated with chl *a* (*p* < 0.001, *r*^2^ = 0.16) and bacterial production (*p* < 0.0001, *r*^2^ = 0.55). A Tukey’s HSD comparison across months confirmed that the decline in richness was significant in January and February (*p* < 0.05). Shannon Diversity and Pielou’s evenness were also significantly lower in January (*p* < 0.0001).

**FIGURE 2:**
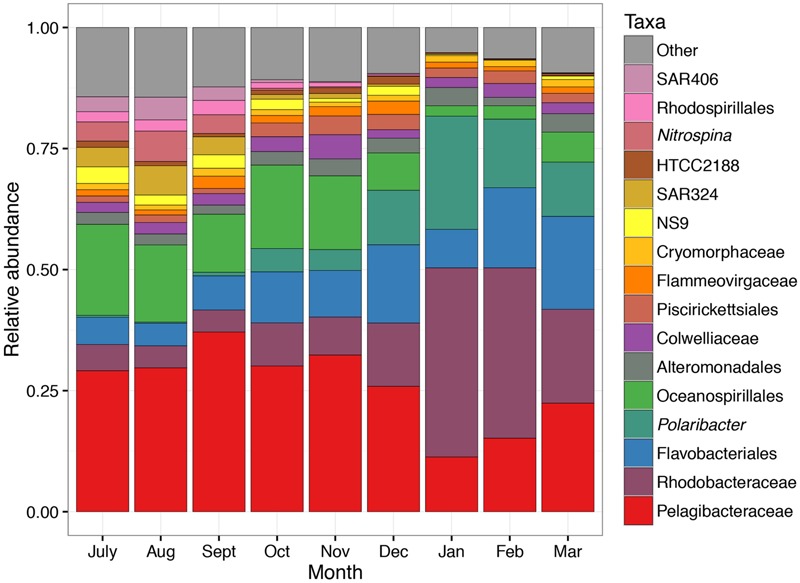
**Taxonomic changes in free-living bacterial community composition over the 9-month sampling period.** Mean relative abundance in each month is shown. Each taxon contains one or more OTUs grouped according to taxonomic assignment to genus level or higher. The lowest taxonomic level available is shown.

**FIGURE 3:**
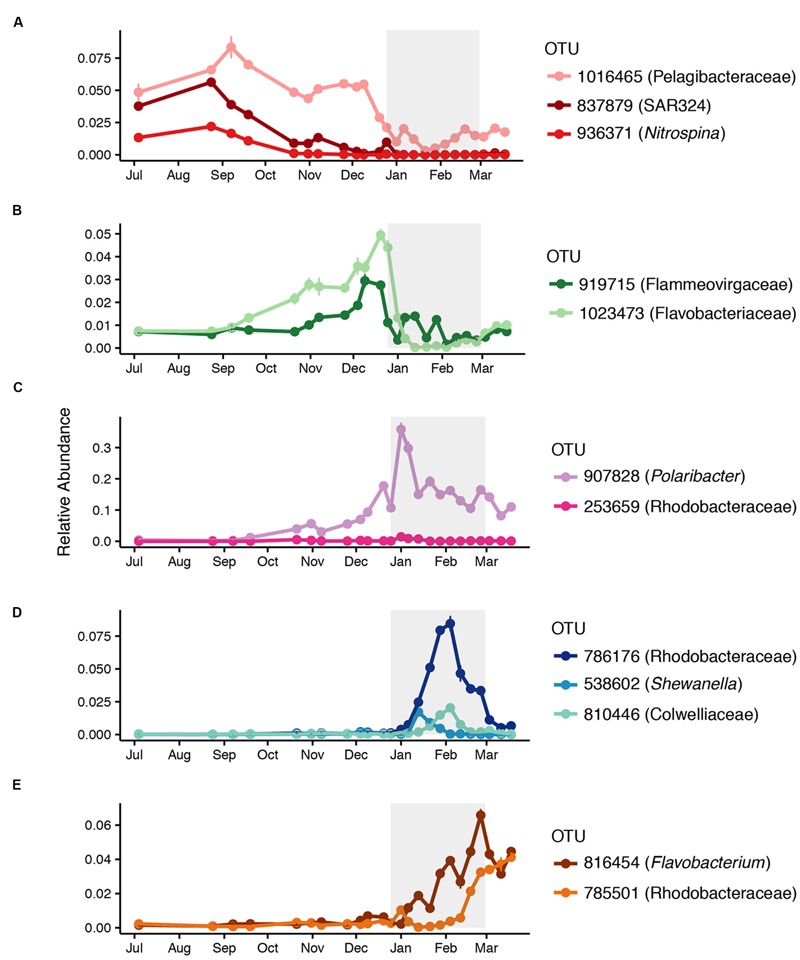
**Successional patterns of free-living OTUs that peaked (A)** during the winter or early spring, **(B)** prior to the summer bloom onset, **(C)** early in the summer bloom, **(D)** in the middle of the summer bloom, or **(E)** at the end of the summer bloom. Mean relative abundance and standard error for each date are shown. The affiliation of each OTU at the lowest taxonomic resolution available is shown.

**FIGURE 4:**
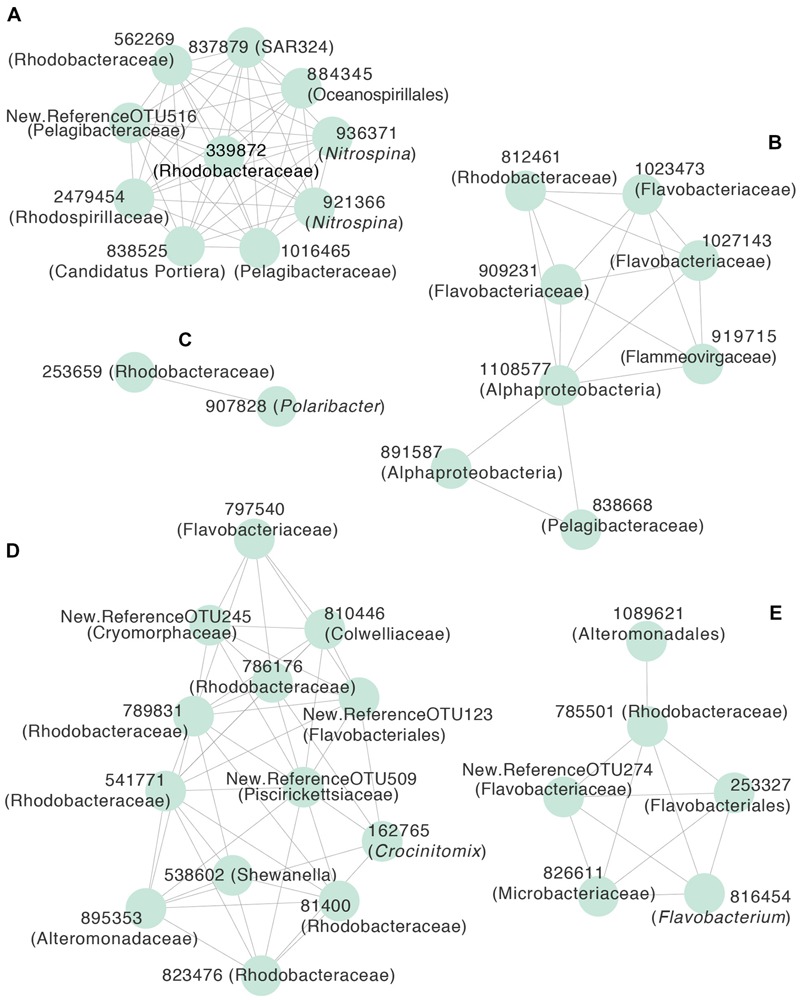
**Correlation network of free-living OTUs with strong temporal patterns in relative abundance, based on Pearson’s correlation (*r* > 0.5) of LOESS-filtered OTUs.** OTUs were partitioned into non-overlapping clusters using *k*-means clustering (*k* = 5). Edges represent positive correlations and nodes represent individual OTUs, labeled with OTU identification number and lowest level of taxonomic resolution available. The letters designate clusters that represented OTUs that peaked **(A)** during the winter or early spring, **(B)** prior to the summer bloom onset, **(C)** early in the summer bloom, **(D)** in the middle of the summer bloom, or **(E)** at the end of the summer bloom. Representative OTUs from each cluster are shown in **Figure 3**.

**Figure 2** provides a broad overview of the changes in relative abundance of free-living bacterial taxa that occurred over the course of the season. Oceanospirillales and Pelagibacteraceae had the greatest relative abundance in winter and spring (July–November). SAR406, Rhodospirillales, and Deltaproteobacteria, including *Nitrospina* and SAR324, were present at greater relative abundances in winter and early spring (July–September) than later in the field season. Changes in the relative abundance of taxonomic groups became most evident in January when Rhodobacteraceae had greater relative abundance than Pelagibacteraceae. *Polaribacter* also increased significantly in January. We did not account for variation in 16S rRNA copy number and thus may have underestimated the abundance of lower copy number taxa like Pelagibacteraceae and overestimated the abundance of higher copy number taxa like Colwelliaceae. In addition to changes in relative abundance for broad taxonomic groups, co-occurrence network analysis with k-mean clustering revealed five temporal pattern types, with individual OTUs that peaked (i) during the winter or early spring, (ii) just prior to the summer bloom, (iii) very early in the summer bloom, (iv) at summer mid-bloom, or (v) after the summer bloom subsided (**Figures 3** and **4**). Some OTUs that were assigned the same family level classification (e.g., Rhodobacteraceae) had distinct temporal patterns corresponding to different times during the summer bloom.

**FIGURE 5:**
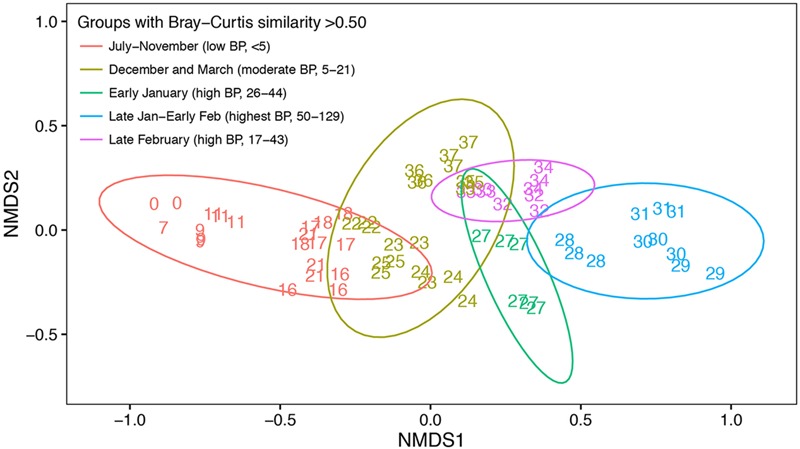
**Non-metric multidimensional scaling of Bray–Curtis similarity indices of free-living bacterial community composition based OTU relative abundance.** Number labels indicate when the sample was collected in weeks since the initial sampling date in July. Identical number labels indicate three replicate samples collected at the same time, except in week 27 (early January) when replicate samples were collected on two separate days. Not every week was sampled during the study period, as indicated by number labels. Color indicates different groups according to hierarchical clustering with a Bray–Curtis similarity cut-off of 0.5. Ellipses represent the 0.95 confidence interval for each Bray–Curtis similarity group. The time period and range of bacterial production (BP) corresponding to each Bray–Curtis similarity group are shown.

NMDS of relative abundances of OTUs demonstrated a shift in community composition from July through December before the onset of the summer phytoplankton bloom (**Figure 5**). Community composition continued to change from December to January as the summer phytoplankton bloom developed (**Figure 4**). March samples clustered midway between the July and November and late January-early February samples, perhaps reflecting the beginning of a return to a pre-bloom community structure. Bacterial communities fell into five groups based on hierarchical clustering of Bray–Curtis similarities. These groups corresponded to different time periods and ranges of bacterial production: July to November (winter and spring; <5 pM l^-1^ h^-1^), December and March (before and after the summer bloom; 5–21 pM l^-1^ h^-1^), early January (beginning of the summer bloom; 26–44 pM l^-1^ h^-1^), late January to early February (summer mid-bloom; 50-129 pM l^-1^ h^-1^), and late February (late in the summer bloom; 17–43 pM l^-1^ h^-1^).

## Discussion

We observed a significant decline in free-living bacterial OTU richness in summer as bacterial production increased during a phytoplankton bloom. Previous studies of temperate systems have also reported richness maxima in the winter and minima in the summer (Gilbert et al., 2012; Ladau et al., 2013). Similar seasonal trends in bacterial OTU richness have been reported in Antarctic waters (Murray and Grzymski, 2007; Ghiglione and Murray, 2012; Grzymski et al., 2012; Luria et al., 2014). Gilbert et al. (2012) observed relatively gradual changes, reporting that day length and serial day alone explained over 66% of the observed variance in richness during a 6-year time series study of the English Channel. Likewise, during a 6-year study in the Northwest Atlantic Ocean, El-Swais et al. (2015) observed a much longer period of decreased richness during the summer and spring that accompanied an extended period of increased primary production. In contrast, we observed only a brief period (∼4 weeks) of decreased richness that coincided with increased bacterial production rates. The abbreviated nature of phytoplankton blooms in the WAP along with high-resolution temporal sampling enabled us to observe this pattern, which contrasts with the multiple annual blooms and/or long periods of sustained growth seen in temperate systems. The results of our study demonstrate close temporal linkages between episodic but biologically significant events like phytoplankton blooms and changes in bacterial production and richness.

In addition to changes in alpha diversity, we measured temporal variation in the taxonomic composition of free-living bacterial communities. The most abundant winter bacterial taxonomic group in our amplicon sequence data was Pelagibacteraceae, a globally abundant clade that correlates negatively with primary production (Morris et al., 2002; Sowell et al., 2009). In addition, several bacterial taxa involved in alternative energetic pathways, e.g., SAR406 (sulfur oxidation; Wright et al., 2013), *Nitrospina* (nitrite oxidation; Spieck et al., 2014), and SAR324 (sulfur oxidation and carbon fixation; Sheik et al., 2014) dominated early in the winter, in accordance with previous suggestions that chemolithoautotrophy is an important metabolism contributing to cellular production during the winter (Manganelli et al., 2009; Grzymski et al., 2012; Williams et al., 2012). Although we did not sequence archaea in this study, ammonia-oxidizing Thaumarchaeota contribute significantly to chemolithoautotrophy during winter, and overall ammonia oxidation rates are higher in winter than summer (Tolar et al., 2016). The seasonality in *Nitrospina* may be related to changes in ammonia oxidation rates.

While species richness did not change significantly until the phytoplankton bloom in summer, free-living bacterial community composition began to change as early as October, with declining relative abundances of Pelagibacteraceae, SAR406, SAR324, and *Nitrospina*. As the season progressed, increasing competition or top down control may have caused declines in the relative abundances of these taxa, while increasing water temperatures and therefore increased water column stratification would have prevented replenishment of taxa from deeper waters. The increase in relative abundance of taxa like Flavobacteriaceae, *Polaribacter*, Flammeovirgaceae, and Rhodobacteraceae before the summer phytoplankton bloom is somewhat surprising given the low levels of chl *a* and bacterial production during the time period. A brief increase in chl *a* in late October coincided with a decline in richness and an increase in bacterial production. While richness increased when chl *a* subsided, bacterial production remained slightly elevated over the next 2 months. These pre-summer changes in community composition could reflect a direct response by certain bacterial taxa to increasing sunlight, in addition to low-level and ephemeral increases in phytoplankton. For example, some marine Flavobacteria, including *Polaribacter*, contain proteorhodopsin, a light-driven proton pump that can enhance growth in low-nutrient conditions, while Rhodobactaceae have been shown to carry out aerobic anoxygenic photosynthesis (Gonzalez et al., 2008; Kimura et al., 2011; Voget et al., 2015; Xing et al., 2015). Whether or not pre-summer changes in bacterial community composition are associated with bacterial feedbacks on phytoplankton growth is not known (Amin et al., 2012, 2015; Prieto et al., 2015; Wang et al., 2016).

As the summer phytoplankton bloom developed, we observed changes in community composition that corresponded to changes in bacterial production. The relative abundance of free-living *Polaribacter* doubled while that of Rhodobacteraceae tripled during the phytoplankton bloom. We noted that different OTUs within Flavobacteriaceae, including *Polaribacter*, and Rhodobacteraceae peaked at different points during the phytoplankton bloom. This suggests niche specialization by different taxa within these groups, perhaps corresponding to different stages in the successive degradation of phytoplankton-derived organic compounds (Teeling et al., 2012; Klindworth et al., 2014). *Polaribacter* occurred in a cluster with only one other OTU (**Figures 3** and **4**), suggesting a strong competitive advantage for *Polaribacter* early in the bloom, in support of the hypothesized role of this taxon (Williams et al., 2013). OTUs of Rhodobacteraceae that peaked later in the bloom may indicate that they utilize secondary products of decomposing organic matter, such as low molecular weight compounds (Voget et al., 2015). We observed a mid-bloom increase in the relative abundance of Gammaproteobacteria, including *Shewanella* and Colwelliaceae, taxa previously associated with substrate rich environments (Baelum et al., 2012; Delmont et al., 2014). Early February peaks in the abundance of OTUs classified as Rhodobacteraceae and Colwelliaceae corresponded to almost complete depletion of nitrate and nitrite, an unusual event in the WAP that might have caused phytoplankton to release carbohydrates (Carlson and Hansell, 2014). It is important to note that our focus on the <3.0 μm fraction of the bacterial community excluded particle-associated bacteria, which may have played an increasingly important role as the phytoplankton bloom developed (Riemann et al., 2000).

We used distributed lag models to statistically test the dynamic relationship between phytoplankton abundance based on chl *a* and bacterial production and abundance during this period. The inclusion of 10- and 20-day lags in our model allowed us to capture a greater portion of the variability in these relationships. In January, peak bacterial production occurred about 20 days after the peak in chl *a*, in agreement with previous studies of Antarctic phytoplankton bloom dynamics (Billen and Becquevort, 1991; Ducklow et al., 2001). A secondary chl *a* peak in mid-January would have presumably also contributed to the peak in bacterial production that occurred about 10 days later. Bacterial reliance on high molecular weight phytoplankton-derived organic matter that must undergo extracellular hydrolysis prior to bacterial uptake is thought to drive such temporal lags (Billen and Becquevort, 1991; Lancelot et al., 1991; Ducklow et al., 2001; Kirchman et al., 2001). Furthermore, some DOM fractions may become available through intermediate trophic processes (e.g., zooplankton sloppy feeding or excretion; Ducklow et al., 2012). The significance of both 10- and 20-day lags may reflect the complex nature of WAP DOM degradation, in which individual bacterial taxa respond within different time frames and specialize in different fractions of the DOM pool (Nikrad et al., 2014; Bowman and Ducklow, 2015; Kim and Ducklow, 2016).

We hypothesize that resource supply from phytoplankton-derived carbon was the primary factor driving the summertime shift in free-living bacterial community composition, although biotic interactions and top–down control by grazing and viral lysis likely modulate the patterns that we observed (Bird and Karl, 1999; Brum et al., 2016). Our measurements did not directly address the underlying mechanisms controlling temporal patterns in bacterial community composition. In particular, we did not directly measure the composition or turnover of organic compounds that we hypothesize contributed to changing bacterial communities. Our study and other similar studies lack a multi-year dimension to be definitive. Nevertheless, the general pattern that we observed is consistent with the view that increased resource supply of a diverse pool of organic compounds that results from phytoplankton blooms plays a strong role in structuring bacterial communities (Teeling et al., 2012; Buchan et al., 2014).

Taken together, our bacterial production, richness, and community composition data indicate strong trophic coupling between bacteria and phytoplankton. Our results agree with previous reports that WAP bacterial density and production are positively correlated with primary production (Church et al., 2003; Ortega-Retuerta et al., 2008), but contrast with earlier reports that the microbial loop is uncoupled from primary producers during spring phytoplankton blooms (Bird and Karl, 1991, 1999; Karl et al., 1991; Duarte et al., 2005). Although community shifts like those we observed suggest strong potential for degradation of labile phytoplankton-derived organic matter (Landa et al., 2014), WAP bacterial standing stocks and productivity are low relative to primary production (∼5% versus 10–20% global average; Ducklow et al., 2012; Kim and Ducklow, 2016). Despite the longstanding debate about the role of temperature in limiting bacterial production in cold water, recent analyses found that temperature is not the principal factor regulating bacterial growth in the Antarctic (Thingstad et al., 1991; Ducklow et al., 2012). However, there is growing evidence, including results from this study, that organic matter availability is a primary factor controlling bacterial growth and community composition in Antarctica (Kirchman et al., 2009; Ducklow et al., 2012; Kim and Ducklow, 2016).

## Author Contributions

CL, LA-Z, HD, and JR designed and conducted the study. LA-Z contributed sequence data for the study and input on analyses and manuscript content. CL analyzed data and wrote the manuscript with guidance from JR. LA-Z, HD, and JR provided input and edited the manuscript. All authors approved the final version of the manuscript.

## Conflict of Interest Statement

The authors declare that the research was conducted in the absence of any commercial or financial relationships that could be construed as a potential conflict of interest.

## Footnotes

1 http://oceaninformatics.ucsd.edu/datazoo/data/pallter/datasets
