# Supplementary material for: Seasonal Succession of Free-Living Bacterial Communities in Coastal Waters of the Western Antarctic Peninsula
Source: Front Microbiol. 2016 Nov 3;7:1731. doi: 10.3389/fmicb.2016.01731 (PMC5093341; doi:10.3389/fmicb.2016.01731)
Supplement: Supplementary file 2 [file Presentation_1.PDF]

Supplemental Text 1. Linear regression of bacterial production and environmental factors

Two data sets were analyzed using linear regression to examine the relationships between bacterial production and environmental factors (EF). One data set was the measured variables, without interpolation. These data were analyzed using simple linear regression of each individual variable in relation to bacterial production (Supplemental Table 1, Measured univariate r^2) (See below for tests with multiple factors). The second data set contained additional interpolated data, needed to create regular time series and enable stepwise linear regression of different lag times between bacterial and environmental factors. Interpolation added data between measured time points; therefore, results were generally more significant in the analysis of interpolated data compared to measured data, as indicated by *p*-values and some cases r^2-values (Supplemental Table 1, compare Measured univariate r^2 to Interpolated univariate r^2). However, results for regression analyses are significant based on a cut-off of $p<10^{-5}$, whether measured or interpolated data were analyzed (Supplemental Table 1).

To analyze the effects of adding different lag times, initial stepwise linear regression equations of the interpolated time-series data followed the form:

Bacterial production ~ (EF with 0-day lag) + (EF with 10-day lag) + (EF with 20-day lag)

Where *EF* is one of the individual variables listed below (Supplemental Table 1). From the initial equation, independent variables (i.e. EF with different lags) were selected based on minimizing Akaike information criterion (AIC). Variables were added and removed in order of greatest reduction in AIC in a stepwise manner (*i.e.* backward elimination and forward selection), thereby seeking an optimal balance between goodness of fit and parameterization. Models with multicollinearity, based on variance inflation factors (VIF) > 5, were discarded.

Final linear regression models were assessed for: 1) fit, 2) significance, 3) VIF, 4) AIC, and 5) parsimony. Based on these criteria, the best model was the distributed lag linear regression model based on chl *a*:

Bacterial production ~ (chl a *with 0-day lag) + (chl* a *with 10-day lag) + (chl* a *with 20-day lag)*

The r^2 of this model is shown in Supplemental Table 1 (see Stepwise r^2). Adding additional lag terms to this equation did not improve the fit based on r^2. However, removing lag terms decreased the fit (e.g., Supplemental Table 1, compare Interpolated univariate r^2 to Stepwise r^2). Although we tested regressions incorporating multiple environmental factors as independent variables, the resulting models did not improve upon the distributed lag model based on chl *a* alone and, furthermore, often displayed multicollinearity.

Supplemental Table 1. Univariate and stepwise linear regressions of bacterial production in relation to environmental factors. Each reported r^2 represents a separate regression analysis.

Environmental factor (EF)	Measured univariate[a] r^2	Interpolated univariate[b] r^2	Stepwise lags[c] (days)	Stepwise r^2
Temperature	0.46	0.59	0, 10	0.68
Phosphate	0.49	0.75	NS[d]	NS
Silicate	0.29	0.53	NS	NS
Nitrate+nitrite	0.52	0.77	NS	NS
POC	0.34	0.62	0, 10, 20	0.79
PON	0.31	0.62	0, 20	0.69
Chl *a*	0.23	0.25	0, 10, 20	0.93

[a]Univariate linear regression of measured variables ($p<10^{-5}$, degrees of freedom (df)=48, except for chl *a* where $p<10^{-7}$ and df=99).
[b]Univariate linear regression of interpolated variables ($p<10^{-15}$, df=162, except for chl *a* where df=234).
[c]Significant lags ($p<0.001$) in the final stepwise linear regression equations based on minimized AIC are shown ($p<10^{-15}$, df=162, except for chl *a* where df=234).
[d]NS is not shown due to significant multicollinearity between different lags (VIF > 5).

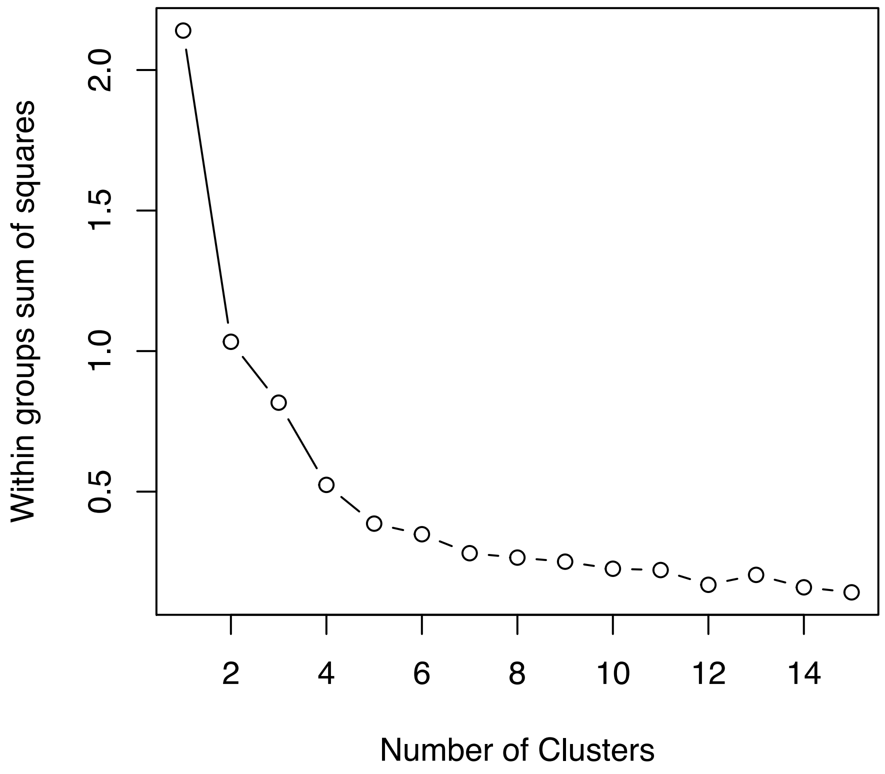

Supplemental Figure 1. Within groups sum of squares with different number of clusters for LOESS-filtered OTUs. Five clusters were visually selected based on the elbow to minimize cluster number and within groups sum of squares.

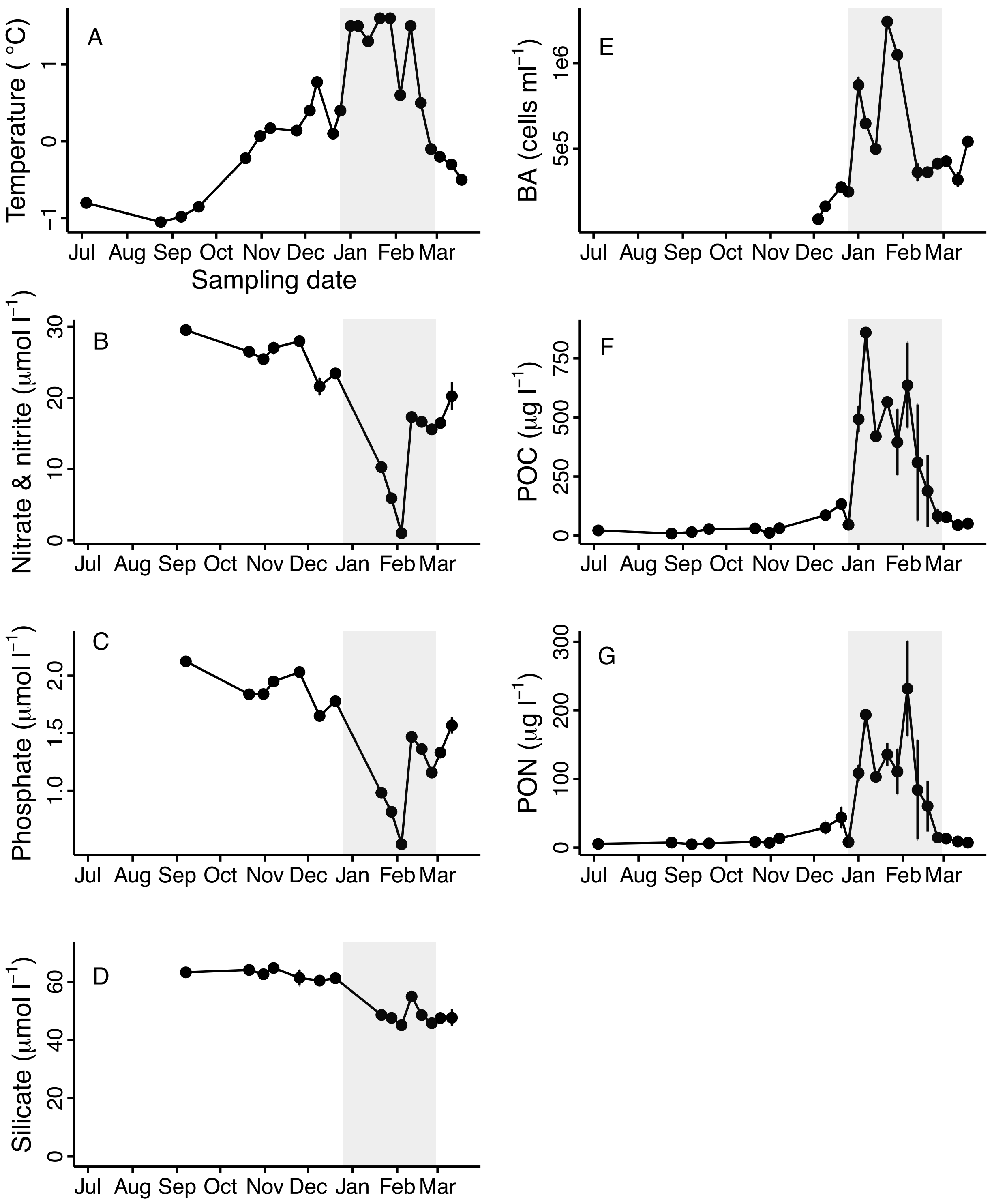

Supplemental Figure 2. Contextual data associated with sequence data including (A) temperature, (B) nitrate and nitrite, (C) dissolved inorganic phosphate, (D) dissolved silicate, (E) bacterial cell abundance (BA), (F) particulate organic carbon, and (G) particulate organic nitrogen. Mean and standard error for each time point are shown.

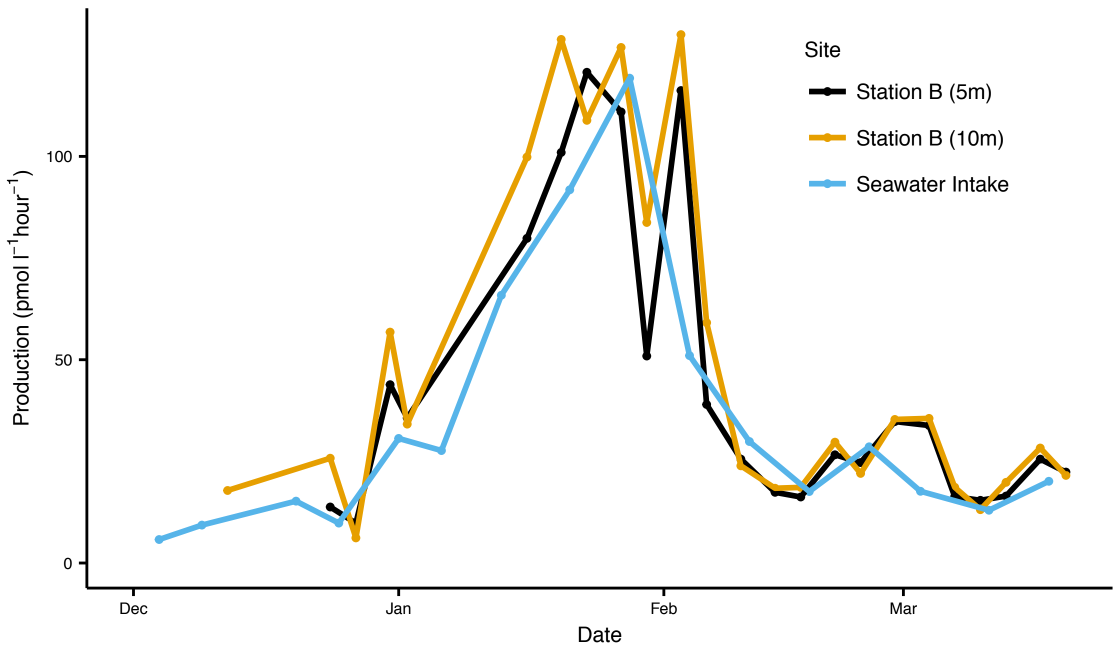

Supplemental Figure 3. A comparison of bacterial production from Palmer Station seawater intake and LTER Station B at 5-m and 10-m depths, December 2013-March 2014.

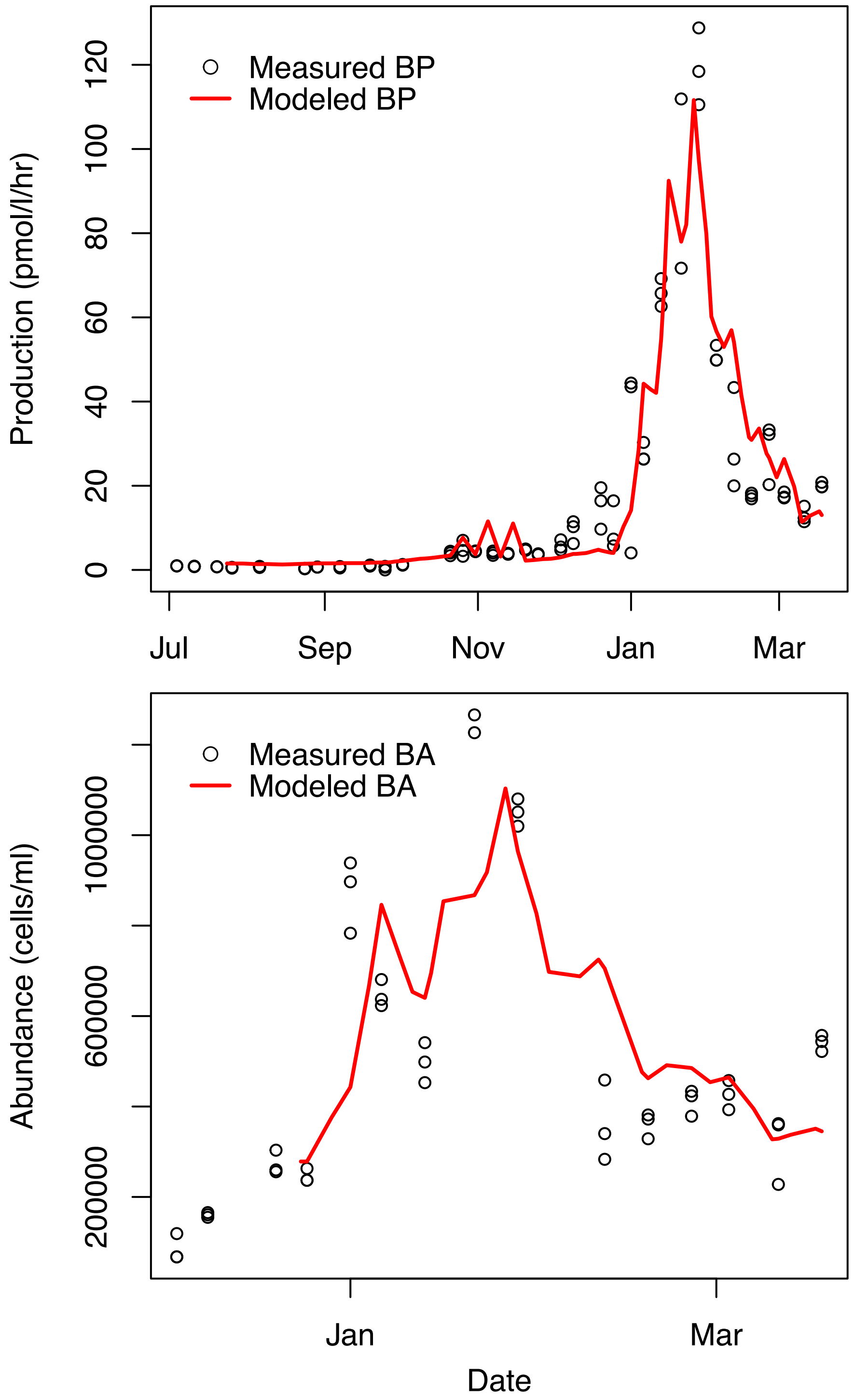

Supplemental Figure 4. Measured and modeled (A) bacterial production and (B) bacterial abundance. Models are based on chlorophyll *a* with 0-, 10-, and 20-day lags as independent variables selected through stepwise linear regression with minimized Aikaike Information Criteria.

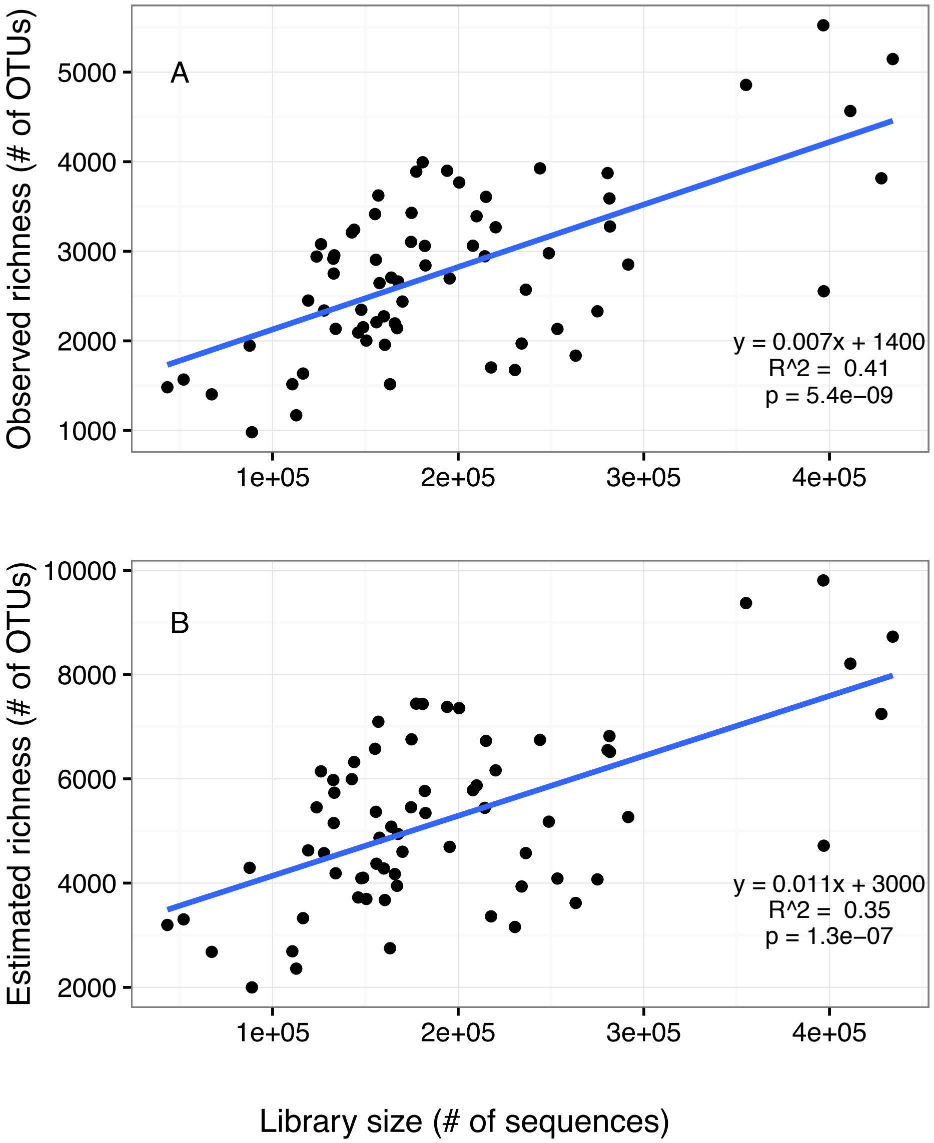

Supplemental Figure 5. Both (A) observed richness and (B) Chao-Jost estimated richness had significant, positive correlations with un-rarefied library size.

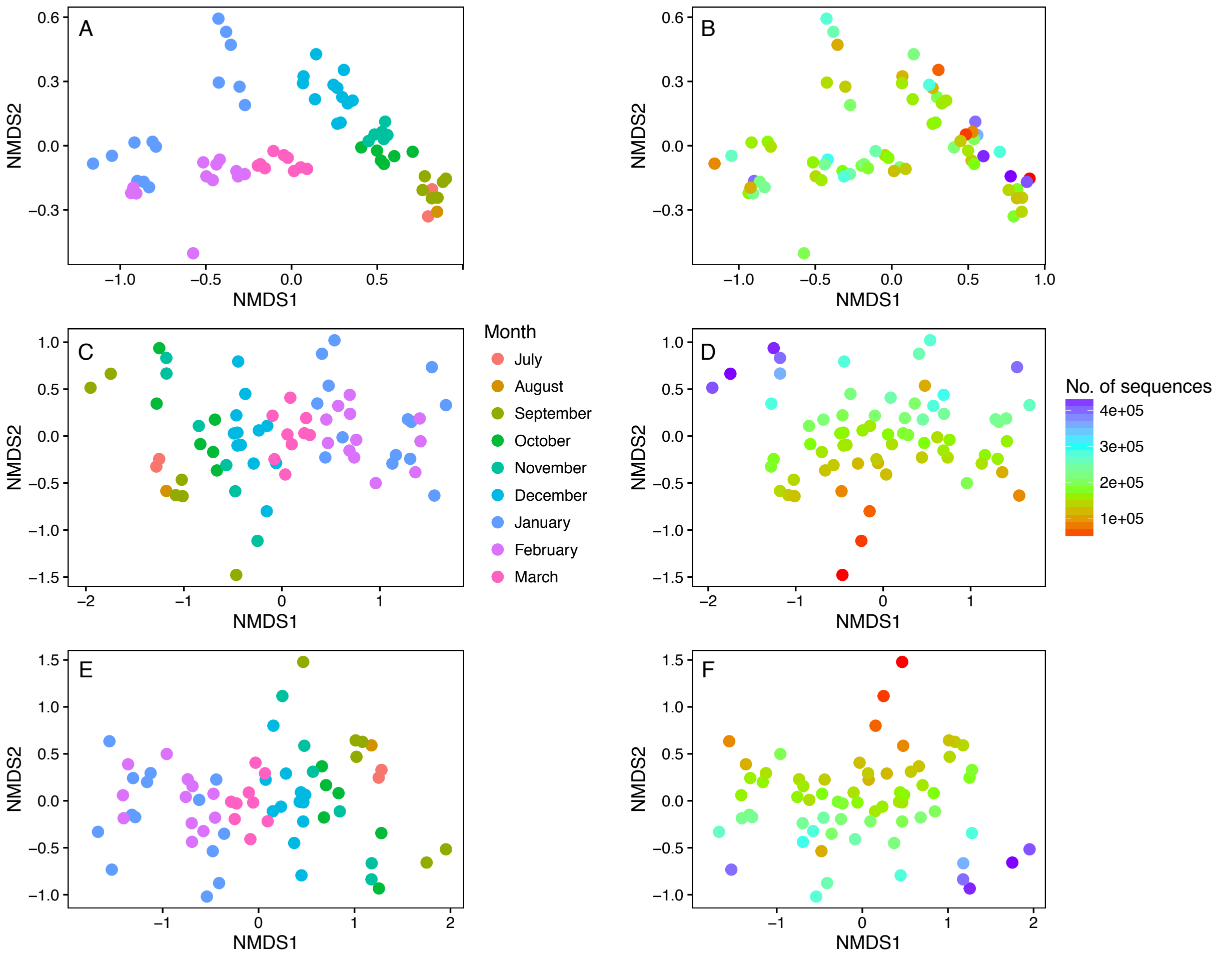

Supplemental Figure 6. Non-metric multidimensional scaling after running three different normalization methods to accommodate size differences between un-rarefied libraries: simple conversion to relative abundance (A-B), Relative Log Expression (RLE) normalization (C-D), and Trimmed Mean of M-Values (TMM) normalization (E-F). Figures in left column contain samples color-coded by month. Figures in right column contain samples color-coded according to library size (number of sequences) prior to normalization.
